# Amaranth (*Amaranthus cruentus* L.) and canola (*Brassica napus* L.) oil impact on the oxidative metabolism of neutrophils in the obese patients*

**DOI:** 10.1080/13880209.2019.1569696

**Published:** 2019-03-24

**Authors:** Dominika Kanikowska, Alina Kanikowska, Rafał Rutkowski, Małgorzata Włochal, Zofia Orzechowska, Aldona Juchacz, Agnieszka Zawada, Marian Grzymisławski, Magdalena Roszak, Maki Sato, Andrzej Bręborowicz, Janusz Witowski

**Affiliations:** a Department of Pathophysiology, Poznań University of Medical Sciences, Poznań, Poland;; b Department of Internal Diseases, Metabolism and Nutrition, Poznań University of Medical Science, Poznań, Poland;; c Department of Computer Science and Statistics, Poznan University of Medical Sciences, Poznań, Poland;; d Departament of Physiology, Aichi Medical University, Nagakute, Japan

**Keywords:** Neutrophil oxidative burst, obesity, plant oil

## Abstract

**Context:** Amaranth and canola oils have been used traditionally. Amaranth has been identified as being of interest because of its outstanding nutritive value. Amaranth oil is a rich source of highly unsaturated fats and so could be a valuable dietary alternative for individuals affected with obesity. Reactive oxygen species (ROS) are postulated to be involved in systemic inflammation and oxidative stress. Activated polymorphonuclear neutrophils (PMNs) generate high amounts of reactive oxygen species.

**Objective:** Our study investigates the impact of amaranth and canola oils supplementation on oxidative metabolism in patients with obesity. We hypothesized that, due to its lipid-lowering and antioxidant properties, amaranth and canola oil would protect against oxidative stress.

**Materials and methods:** We tested 19 obese patients [body mass index (BMI) = 41.1 ± 7.8 kg/m^2^, (mean ± SD)]. The protocol consisted of two stages: a run-in phase of 2 weeks and an experimental stage – canola or amaranth oil supplementation (20 mL/d) with calorie restriction diet for 3 weeks. The neutrophil oxidative burst was expressed by fluorescence intensity (IF).

**Results:** The oxidative burst had increased significantly at the end of treatment in both groups IF: (21.4 ± 11.15 vs. 35.9 ± 20.3; mean ± SD) *p <* 0.05. The levels of IF were significantly higher in neutrophils of patients who received canola oil (41.05 ± 25.3) compared to those who received amaranth oil (28.4 ± 11.8) *p* < 0.05.

**Conclusions:** Canola oil exerts possible effects on oxidative burst activity in neutrophils *in vivo* conditions.

## Introduction

Oxidative stress is the result of an imbalance between the production of reactive oxygen species and an antioxidant mechanism. Reactive oxygen species are hypothesized to be involved in the systemic inflammation and oxidative stress in patients with obesity. Adipose tissue consists of adipocytes, but also contains other cells such as pre-adipocytes, lymphocytes, macrophages, fibroblasts and vascular cells; it is mainly found in subcutaneous and visceral depots (Ouchi et al. 2011). Neutrophils are involved in the modulation of adipose tissue inflammation in the early stage of obesity (Talukdar et al. 2012; Huh et al. 2014; Xu et al. 2015).

Obesity is defined as excesses of adipose tissue and is a significant risk factor for metabolic and cardiovascular disease, including type 2 diabetes (Klein et al. 2004; Yach et al. 2006; World Health Organization 2011). Obesity is associated with dysfunction of adipose tissue, including elevated pro-inflammatory markers (Hotamisligil 2006), and these inflammatory factors are major contributors to the development of metabolic disease (Xu et al. 2015). Obesity induces alterations in adipose tissue; a chronic, low-grade systemic inflammatory state is characteristic of obesity that has evolved over a period of time (Lumeng and Saltiel 2011). The inflammatory response associated with obesity results in an increase in circulating cytokines (Hotamisligil et al. 1995) and oxidative stress (Gregor and Hotamisligil 2011).

Obese patients exhibit significant increases in both neutrophil-derived proteins, including myeloperoxidase and calprotectin (Nijhuis et al. 2009; Olza et al. 2012) or neutrophil elastase (Talukdar et al. 2012). Activated polymorphonuclear neutrophils generate high amounts of reactive oxygen species. Increased neutrophil oxidative burst activity has been shown by energy restriction (Suzuki et al. 2003).

It has been established that both reduction in animal fat consumption and increased use of unsaturated fats are beneficial in lowering body weight, which may reduce the risk of cardiovascular disease.

Amaranth (*Amaranthus cruentus* L.) oil contains tocotrienols and squalene compounds (which are known to affect cholesterol synthesis in humans), while canola (*Brassica napus* L.) oil is rich in both α- and γ-tocopherol, which have antioxidative activities (Qureshi et al. 1996). Amaranth oil was found to lower cholesterol in hamsters and chickens (Qureshi et al. 1996; He et al. 2002) as well as in humans (Martirosyan et al. 2007). Canola oil contains monounsaturated fatty acids (MUFAs), polyunsaturated fatty acids (PUFAs) including 61% oleic acid, 21% linoleic acid, and 11% α-linolenic acid (ALA), plant sterols and high concentrations of phytosterols (769 mg/100 mg canola oil); all of which have been shown to be cardioprotective substances (Gunstone 2011). A study by Gillingham et al. (2011) also showed a positive correlation between MUFAs and cardiovascular health through the regulation of plasma lipids and lipoproteins, low-density lipoprotein (LDL) oxidation and insulin sensitivity. Phytosterols can reduce LDL cholesterol by reducing its absorption (Suhad et al. 2008) and have anti-inflammatory and anti-oxidative properties (Szymańska and Kruk 2007). Kim et al. (2006) showed that amaranth oil supplementation in diabetic rats could reduce oxidative stress that was due to the improved endogenous production of superoxide radicals, even though the mechanism underlying the protective action of amaranth oil against oxidation remains unknown. In a study by Anilakumar et al. (2006), consumption of amaranth leaves by rats reduced oxidative stress in their livers, an effect that was attributed to the positive effects of the carotenoids, chlorophyllin and polyphenolic compounds that were present in the leaves. Carotenoids are known to scavenge free radicals and other oxidants (Sies and Stahl 1995). The study by Pasko et al. (2011) confirmed that amaranth grains reduced oxidative stress. This effect was obtained due to the significant amount of the flavonoid rutin in amaranth grains, which contribute to antioxidant activity. In this respect, plant oils (amaranth and canola oils), as a rich source of highly unsaturated fats, could be a potential dietary option for individuals affected by obesity.

Our study investigates whether amaranth and canola oil supplementation impacts oxidative metabolism in patients with obesity during calorie restriction. The hypothesis is that owing to its lipid-lowering and antioxidant properties, amaranth and canola oil will improve neutrophil function (as reflected by the oxidative burst) from individuals who have been receiving amaranth/canola oil supplementation in their diet.

## Materials and methods

Nineteen obese patients [age 48.3 ± 16 years, 6 women and 13 men, BMI = 41.1 ± 7.8 kg/m^2^, (mean ± SD)] were studied. The inclusion criteria were based on body mass index (BMI)>30 kg/m^2^. The exclusion criteria were: nonsmokers, history of cancer, autoimmune diseases, pregnancy, psychiatric disorders, ongoing therapy with antibiotics and/or steroid and alcohol and drug addiction. The study was approved by the Ethics Committee of Poznan Medical University and adhered to basic Principles of the Declaration of Helsinki (NCT03346421 retrospectively registered at 14 November 2017). For all of the patients, the protocol consisted of two stages: a run-in phase of 2 weeks (patients on a standard diet) and an experimental stage – canola oil supplementation (20 mL/d) or amaranth oil supplementation (20 mL/d) with calorie restriction diet for 3 weeks. Testing at an experimental (week 0) and completion stage (week 3) included bioimpedance measurement (Tanita MC 980 MA, Tokyo, Japan) and blood sampling. The dose of 18 ml per day of amaranth oil was chosen based on the study by Martirosyan et al. (2007). The hypocaloric diet was based on a 25–30% reduction in caloric intake compared to total energy requirement. The total daily energy requirement was calculated on the actual body weight using the Harris-Benedict formula and the physical activity level index. To accurately determine the total energy expenditure and to assess the energy value of the diet before the program, the subjects completed a 3-day intake measurement before the study (24 h dietary interview includes 3 days). Subjects received the same type of diet prepared by a caterer. Each patient received a diet with an identical composition of macronutrients (20% calories from protein, 25–30% from fat and 50–55% from carbohydrates). The daily fibre intake was above 25 g.

**Figure 1.**
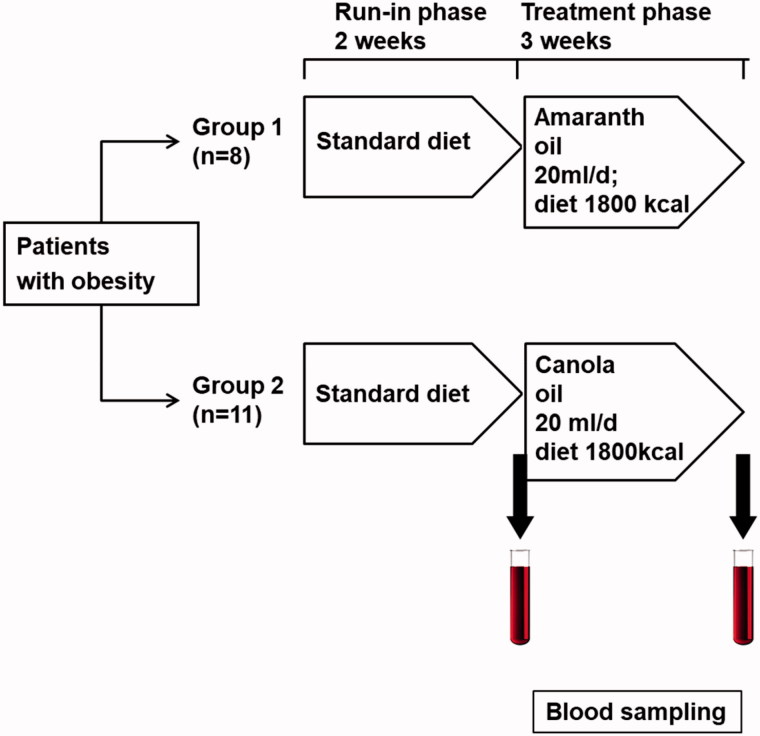
Study protocol.

The patients were placed randomly into two groups- 11 patients in the canola oil and 8 in the amaranth oil group. The supplements were given by commercially available oils (Ol’Amar, Co., Łomża, Poland and VitaCorn Co., Poznań, Poland). The oils were given to patients at the same time of day (mid-morning) to avoid circadian influences, and a dietician supervised their consumption. The study protocol is as follows (Figure 1).

Blood samples (2.5 mL) were collected in lithium heparin tubes. (1) To measure the intensity of ROS production, dihydrorhodamine (123 DHR, Sigma, USA) was added to all the samples at a concentration of 10 µg/mL and incubated in the dark (37 °C) for 5 min. (2) A stimulator of the oxidative burst, phorbol 12-myristate 13-acetate (PMA, Sigma-Aldrich, USA), was added to the samples at a concentration of 40 μg/mL. The samples were then incubated in the dark and at the room temperature for 15 min. (3) 450 µL of red blood cell lysis solution was added to the samples.

Two aliquots of blood were stained with DHR (an uncharged ROS indicator measurable by flow cytometry when oxidized to cationic rhodamine 123). One of the aliquots was stimulated with PMA (a commonly used general purpose laboratory agent that induces cell activation of granulocytes). The mean fluorescence intensity of both the stimulated and unstimulated tubes was measured. The results were expressed as the fluorescence intensity (IF) ratio: IF DHR + PMA/IF DHR. ROS production was measured by BD FACSAria III (BD Biosciences, USA) cytometer and all the results were analyzed using the BD FACSDiva Soft wave (BD Biosciences, USA).

Statistical analysis was performed using the Statistica 12.5 (StatSoft, Tulsa, USA). Values were expressed as mean ± SD and were compared using the Wilcoxon test and Student’s *t*-test, while for the correlation between different parameters, the Spearman correlation coefficient was used. Significance was established at *p* < 0.05.

## Results

**Figure 2.**
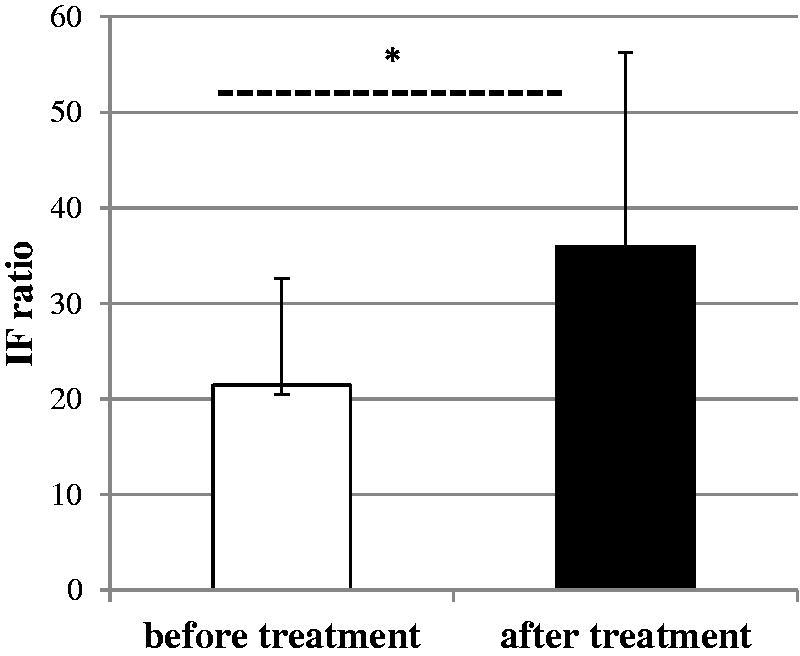
The oxidative burst of neutrophils from obese subjects before and after dietary treatment. The results were expressed as the fluorescence intensity (IF) ratio. ±SD. *p* < 0.05.

The neutrophil oxidative burst expressed as the fluorescence intensity (IF) before and after oil supplementation and caloric restriction treatments are shown in Figure 2. The oxidative burst had increased significantly at the end of treatment in both groups *p* < 0.05.

**Figure 3.**
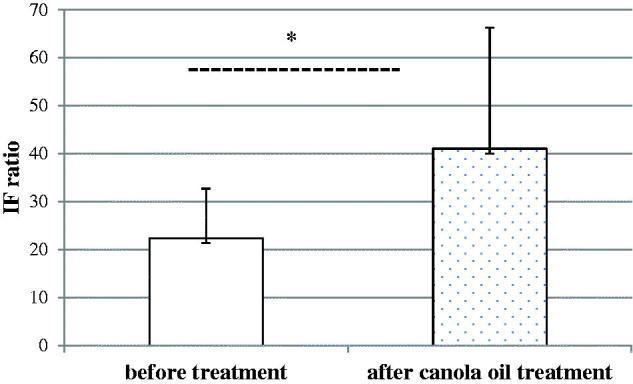
The effect of dietary treatment of canola oil on oxidative burst of neutrophils. The oxidative burst of neutrophils before and after canola oil treatment. The results were expressed as the fluorescence intensity (IF) ratio. ±SD. *p* < 0.05.

**Figure 4.**
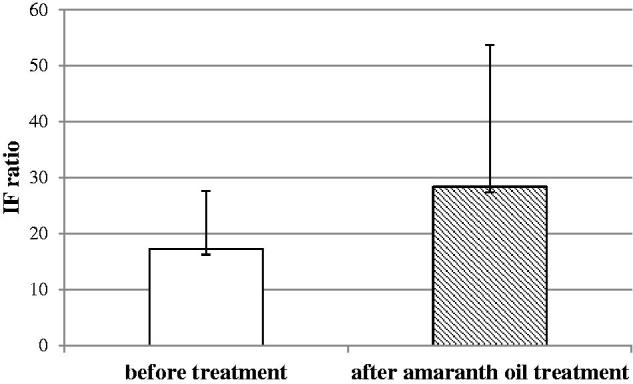
The effect of dietary treatment of amaranth oil on oxidative burst of neutrophils. The oxidative burst of neutrophils before and after amaranth oil treatment. The results were expressed as the fluorescence intensity (IF) ratio. ±SD. *p* > 0.05 (not significant).

The oxidative burst in the group with canola oil supplementation changed significantly (Figure 3), and this significant change was not observed in amaranth oil supplementation group (Figure 4). In Table 1 is shown the changes in weight and composition of the body. In the baseline condition, there was no significant difference in age, weight, and BMI. The body weight before and after oil supplementation and caloric restriction changed significantly in both groups *p* < 0.05. The other parameters: waist/hip ratio, body fat mass, fat-free mass and basal metabolic rate changed significantly only in canola oil group *p* < 0.05.

**Table 1.** Characteristics of the subjects in the two intervention groups (*n* 19). Between-group comparisons were performed using Student’s *t*-test with significance established at *p < 0.005.

	Canola oil (*n* 11)		Amaranth oil (*n* 8)	
	Baseline	After 3 weeks	Baseline	After 3 weeks
Age (years) (±SD)	51.1 (18.02)		43.9 (11.16)	
Height (cm) (±SD)	177.6 (7.42)		171.3 (9.01)	
Weight (kg) (±SD)	126.2 (15.89)	120.5* (15.7)	126.6 (27.66)	118.8* (24.7)
BMI (kg/m^2^) (±SD)	39.9 (4.03)	38.7 (4.8)	42.9 (10.39)	40.3 (9.9)
Waist/hip ratio (±SD)	1.0 (0.10)	1.01* (0.1)	0.9 (0.1)	0.9 (0.1)
Body fat mass (kg) (± (9.7)			49.4 (14.0)	45.7 (19.2)
Fat free mass (± SD)	78.45 (8.8)	75.79* (9.5)	76.56 (16.1)	71.6 (11.2)
Basal metabolic rate (± SD)	2402.182 (309.33)	2312.364* (302.93)	2378.1 (547.8)	2210 (382.0)

Oxidative burst at the baseline and at the endpoint was checked for Spearman correlation with the following covariates: (endpoint value- baseline value) for weight (*r* = −0.23, *p* = 0.33), BMI (*r* = −0.3, *p* = 0.2), waist/hip ratio (*r* = 0.44, *p* = 0.06), body fat mass (*r* = 0.07, *p* = 0.76), fat free mass (*r* = −0.3, *p* = 0.2), and basal metabolic rate (*r* = −0.32, *p* = 0.16). No relations were observed.

## Discussion

The present study was performed to compare the effect of two different dietetic interventions on neutrophil function (as reflected by oxidative burst). In the study, neutrophils from obese individuals after treatment had a significantly higher oxygen production compared to those 3 weeks of dietary treatment (*p* < 0.05) (Figure 2). Suzuki et al. (2003) suggested that oxidative burst activity is affected by energy intake restriction. Similar, a study by Koizumi et al. (1987) showed the modulation of antioxidant enzymes by dietary restriction. The analysis in the current study revealed that body weight and body fat reduction were not correlated with oxidative burst activity at the end of the trial. Therefore, the higher fluorescence intensity in neutrophils we observed in the obese individuals after treatment might be linked to oils treatment. The levels of the fluorescence intensity were significantly higher in neutrophils of patients who received canola oil compared with those who received amaranth oil (Figures 3 and 4). In human clinical trials, there are a limited number of studies with two different oils tested. Cotelle (2001) and Amarowicz and Pegg (2017) show that the food rich in dietary phenolic compounds, which have antioxidant activity, can act as reducing agents, hydrogen-donating antioxidants, could diminish the amount of LDL oxidation *in vivo*, due to the redox properties of plant polyphenols. Interestingly, the antioxidants present in plant-derived oils are capable of interfering with the processes involved in oxidative burst of neutrophils. Furthermore, the studies by Hertog et al. (1995) and Hung et al. (2004) show that the dietary intake and the therapeutic use of phytochemicals may have positive health effects.

Our study is the first to document the possible modulation of canola oil on oxidative burst activity in neutrophils *in vivo* conditions. The present result is in agreement with other studies that found enhanced oxidative burst in healthy young men after 8 weeks of fish oil supplementation (Gorjão et al. 2006; Bartelt et al. 2008). Canola oil is characterized by high levels of PUFAs (Lin et al. 2013). The literature on the effects of dietary supplementation with omega-3 PUFAs on leukocyte functions is not entirely in agreement. Numerous studies have shown that dietary supplementation with omega-3 PUFA results in the reduction of chemotactic migration in neutrophils (Sperling et al. 1993), increased superoxide anion generation (Chen et al. 1994). On the other hand, several experiments have demonstrated the opposite effects on neutrophil function: decreased superoxide anion generation in neutrophils (Poulos et al. 1991). The mechanisms by which PUFAs exert their immune-modulating actions are unknown. The immunosuppressive effect is thought to work through changes in eicosanoid production (Kelley et al. 1999). It has also been suggested that PUFA mediate their effect by changes in gene expression or membrane structure (Szekely et al. 2007).

As canola plant contains high amounts of natural antioxidants, it may exert protective effects through inhibition of ROS production. The study of canola plant by Vu et al. (2015) showed that the higher endogenous flavonol levels correlated with higher ROS scavenging activities.

Our findings suggested a complex and multi-level regulatory system. Further investigations are required to determine the roles of *Brassica napus* on neutrophil function.

In conclusion, the present study suggests an immuno-stimulating effect on oxidative burst after 3 weeks of canola oil-supplementation in obese patients.

## Data Availability

The data supporting the conclusions of this article are included within the manuscript. The dataset is available from the corresponding author on request.
